# Melanogenesis Connection with Innate Immunity and Toll-Like Receptors

**DOI:** 10.3390/ijms21249769

**Published:** 2020-12-21

**Authors:** Saaya Koike, Kenshi Yamasaki

**Affiliations:** 1 Shiseido Global Innovation Center, Kanagawa 220-0011, Japan; saaya.koike@shiseido.com; 2 Department of Dermatology, Tohoku University Graduate School of Medicine, Miyagi 980-8574, Japan

**Keywords:** melanosome, melanogenesis, toll-like receptor, innate immunity, RAB, microphthalmia-associated transcription factor, tyrosinase, Hermansky–Pudlak syndrome, Griscelli syndrome

## Abstract

The epidermis is located in the outermost layer of the living body and is the place where external stimuli such as ultraviolet rays and microorganisms first come into contact. Melanocytes and melanin play a wide range of roles such as adsorption of metals, thermoregulation, and protection from foreign enemies by camouflage. Pigmentary disorders are observed in diseases associated with immunodeficiency such as Griscelli syndrome, indicating molecular sharing between immune systems and the machineries of pigment formation. Melanocytes express functional toll-like receptors (TLRs), and innate immune stimulation via TLRs affects melanin synthesis and melanosome transport to modulate skin pigmentation. TLR2 enhances melanogenetic gene expression to augment melanogenesis. In contrast, TLR3 increases melanosome transport to transfer to keratinocytes through Rab27A, the responsible molecule of Griscelli syndrome. TLR4 and TLR9 enhance tyrosinase expression and melanogenesis through p38 MAPK (mitogen-activated protein kinase) and NFκB signaling pathway, respectively. TLR7 suppresses microphthalmia-associated transcription factor (MITF), and MITF reduction leads to melanocyte apoptosis. Accumulating knowledge of the TLRs function of melanocytes has enlightened the link between melanogenesis and innate immune system.

## 1. Introduction

Melanocytes play unique roles in the production of melanin, a fundamental molecule of skin pigment. Melanocytes synthesize melanin pigments by receiving various external stimuli such as ultraviolet rays and peptide hormones melanocortins. Melanocytes deliver melanin to the adjacent epidermal keratinocytes, and then the transported melanin functions as a UV filter in the keratinocytes. Thus, melanocytes and melanin play roles in the biological defense of human epidermis. Melanocytes are also involved in innate immunity, which conducts the initial responses in the elimination of microorganisms and viruses. Melanocytes augment melanogenesis and melanin transport by innate immune stimuli through toll-like receptors (TLRs). These findings suggest that melanin synthesis and melanin transport have connections with the immune systems. In this review, we provide an overview of the skin functions in innate immune systems and review the melanocyte functions in immune responses and pigment formation induced by innate immunity in order to discuss the significance of innate immunity in melanogenesis and skin pigmentation.

## 2. Biological Defense Mechanism by Adjusting the Color Tone of Epidermal Cells

The skin is the outermost organ of the human body and functions as the main defense system. The skin maintains the human homeostasis by separating the internal environment from the external environment [1]. The epidermis is located at the surface of the skin and is in continuous contact with external stimuli such as dryness, ultraviolet rays, and microorganisms. To protect the internal organs, epidermal cells have various biological defense functions [2]. The physical barrier function of the epidermis plays a major role in protection against infections. The lipid layer on the epidermis, the cornified layer of keratinocytes, and tight junctions in the granular layer are the major physical barrier of epidermis. Skin pigment has an essential function in protecting the body from the surrounding environment such as UV irradiation. Adjusting skin color has a biological defense function for animals, such as camouflage to hide from foreign enemies. Since the innate immune system detects and responds to changes in the surrounding environment, it is reasonable to assume that the innate immune system is involved in the regulation of skin color in epidermal cells.

### 2.1. Keratinocytes

Keratinocytes, which make up approximately 90% of epidermal cells, have a skin barrier function. Keratinocytes form the structure of the stratum corneum, which is a layer made by a process called keratinization when keratinocytes become mature, moving toward the upper layer. The stratum corneum structure serves as a physical barrier [1]. Keratinocytes not only physically protect the human body but are also involved in immune functions in the skin. Keratinocytes serve as the first line to detect pathogens and to induce defensive molecules against pathogens. Epidermal keratinocytes express TLRs and elicit an immune response [3]. The roles of epidermal TLRs in innate immunity have been actively studied [4,5,6]. Epidermal cells directly interact with specific molecular structures, pathogen-associated molecular patterns (PAMPs) of microorganisms and viruses. PAMPs include bacterial cell wall peptidoglycans, Gram-positive bacterial lipopeptides, Gram-negative bacterial lipopolysaccharides, and viral double-stranded RNA. When TLRs recognize PAMPs, keratinocytes activate NFκB and other intracellular signals to enhance the immune responses and elicit cytokines, chemokines, and the production of antimicrobial peptides [7].

Keratinocytes also play an important role in the expression of skin color. Keratinocytes protect the human body from UV damage by taking in melanosomes delivered from melanocytes and aggregating them around the nucleus. In addition, keratinocytes themselves strongly induce melanin synthesis by releasing several factors. Keratinocyte promotes melanogenesis, melanocyte proliferation, and dendrite formation of melanocytes through the production and release of factors such as the proopiomelanocortin (POMC)-derived peptides α- Melanocyte- Stimulating Hormone (α-MSH) and adrenocorticotropic hormones (ACTH), stem cell factor (SCF), endothelin-1 (ET-1), which are stimulated by UV rays [8]. TLR2 ligand lipoteichoic acid (LTA) induces the release of the stem cell factor (SCF) of keratinocytes [9]. Since SCF promotes melanin synthesis in melanocytes by activating microphthalmia-associated transcription factor (MITF) transcription via SCF/c-KIT signaling [10], TLR2-mediated innate immune stimulation would regulate melanin synthesis by inducing cytokines from keratinocytes.

### 2.2. Melanocytes

Melanocytes are localized in the basal layer of the human epidermis. Melanocytes produce a pigment molecule, melanin, and form a chemical barrier against UV for protection of the skin. Melanin is synthesized in the melanocyte-specific organelle melanosome. Melanocytes deliver the melanin synthesized in melanosomes to neighboring keratinocytes. The delivered melanosomes aggregate above the nucleus in keratinocytes and form a structure called the ‘melanin cap’. The melanin cap absorbs ultraviolet rays and reduces DNA damages caused by UV photoproducts. Thereby, melanin prevents sun damage and skin carcinogenesis [11]. Melanin decreases UV-induced DNA photoproducts in the epidermis in a dose-dependent manner [11]. Melanin also suppresses free radical energy generation by sunlight exposure. Melanin absorbs free radical energy and converts it into heat, reducing cell damage otherwise caused by oxidative stress [12]. The importance of the skin pigment in skin carcinogenesis has been well documented. A study of nonmelanoma skin cancer in the USA found that people with darker skin are less susceptible to sun-induced skin cancer than people with lighter skin pigment [13]. Darker skin has much more melanin than lighter skin, and the melanosome shape also differs between darker and lighter skin. Darker skin melanosomes are elliptical and evenly distributed, and they are tightly stacked above the nucleus to form a strong melanin cap sunscreen. Lighter skin melanosomes, however, are roundly aggregated and can form only a poor melanin cap sunscreen [14]. In addition to functions in UV protection, melanin pigment is involved in various aspects of epidermal biological defense, such as metal adsorption, drug uptake, thermoregulation, and camouflage to avoid external enemies [14]. Thus, melanocytes function as a part of the physical defense mechanism in the skin barrier.

## 3. Melanocyte Functions in Immune System and Inflammation

### 3.1. Melanocyte Functions in Acquired Immunity

In epidermal cells, melanocytes and Langerhans cells are morphologically classified as dendritic cells. As professional antigen presenting cells (APCs), Langerhans cells as well as dermal dendritic cells have particularly strong antigen presenting ability. APCs take up a foreign antigen into cells by endocytosis and phagocytosis and present the antigen peptides. APCs express a glycoprotein called MHC class II and present the antigen to T cells by forming a complex with foreign antigen peptides. Although melanocytes are not recognized as a professional APCs, melanocytes express MHC class II by IFNγ stimulation [15,16,17]. The ability for phagocytosis is also observed in melanocytes. Le Poole et al. documented that melanocytes had phagocytosis ability, which was examined using 1 µm latex beads in the presence of keratinocytes [18]. Le Poole et al. also demonstrated that melanocytes can function as target cells for T cells by processing and presenting the phagocytosed antigen [15]. Melanocytes express intercellular adhesion factor ICAM-1, which is responsible for the cell-cell interactions of the immune system, and CD40, which is mainly observed in mature dendritic cells and plays roles in adaptive immune systems [17,19,20,21]. In a manner similar to that of the professional APCs, melanocytes also produce cytokines that modulate immune responses, such as IL-1α, IL-1β, IL-8, and TGF-β1 [22,23,24]. These finding suggest the possible roles of melanocytes as antigen-presenting cells.

### 3.2. Melanocytes and Immunodeficiency Disorders

Albinisms, hypopigmentary disorders, are caused by a genetic alteration of the melanin synthetases and melanosome-related molecules. Some albinisms are complicated by disorders of immune functions, suggesting genetic relationships between melanin production and immune function.

**Figure 1:**
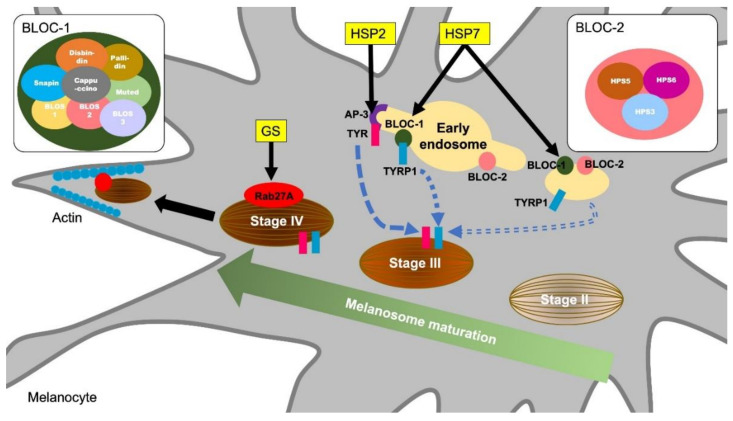
Molecular mechanisms of melanosome formation and maturation related to the hypopigmentary genetic diseases. Griscelli syndrome (GS) is an autosomal recessive disorder characterized by immunodeficiency and partial bleaching due to abnormal melanosome distribution. One of the causes of GS is a mutation in the Rab27A gene. Rab27A transports melanosome to the cell periphery, and Rab27A gene mutations result in a melanosome transport disturbance in melanocytes. Hermansky–Pudlak syndromes (HPS) are also rare autosomal recessive disorders and are associated with skin depigmentation and immune impairment. HPS2 patients have a deficiency in the β3A subunit of AP-3. AP-3 classifies the melanin synthesis-related enzyme tyrosinase (TYR) from endosomes to melanosomes. Patients with HSP7 do not express the dysbindin protein, one of the components of BLOC-1 (biogenesis of lysosome-related organelles complex 1). BLOC-1 regulates the transport and biosynthesis of lysosomal organelles. BLOC-1 interacts with AP-3 to promote the transport of tyrosinase-related protein 1 (TYRP1). BLOC-1 also interacts with BLOC-2 and promotes TYRP1 transport by a mechanism different from that of AP-3.

Griscelli syndromes (GS) are autosomal recessive disorders characterized by immunodeficiency and partial depigmentation due to aberrant melanosome distribution [25]. Rab27A is one of the genes responsible for causing GS [26]. Because Rab27A is a regulator of intracellular membrane trafficking and is essential for melanosome transport to the dendrite tip in human melanocytes, a defect of the Rab27A function causes the depigmentation of skin observed in GS [27] (Figure 1). Rab27A is also involved in immune functions. Individuals with the Rab27A mutation develop lymphocyte and macrophage activation syndrome. Rab27A-deficient T cells have reduced cytolytic granule exocytosis, which is caused by a defect of intracellular membrane trafficking [26]. These findings suggest that Rab27A is an important effector of the cytotoxic granule transport, which is an essential pathway for immune homeostasis.

Hermansky-Pudlak syndromes (HPS) are autosomal recessive disorders known to be associated with depigmentation and immune disorders [28,29]. Defects in cytoplasmic organelles such as melanosomes, platelet granules, and lysosomes cause ocular cutaneous albinism, bleeding tendency, and ceroid lipofuscin lysosomal storage disease [29]. Pigment abnormalities in HSPs are caused by a decrease in the number of melanosomes and increase in immature melanosomes in melanocytes [30]. Some types of HPS result from mutations in genes encoding endoplasmic reticulum transport proteins, which are involved in the biosynthesis of organelles containing melanosomes [31]. HPS2 is caused by a deficiency in the β3A subunit of the adaptor protein-3 (AP-3) complex [32]. The AP-3 complex controls sorting of the protein cargo into specialized organelles, including endosomes, lysosomes, and melanosomes [33]. Microbial antigens phagocytosed by APCs undergo antigen binding to MHC and CD1 molecules after being delivered to the lysosomal compartment. However, in AP-3 deficient cells derived from HPS2 patients, CD1b does not have efficient access to lysosomes, resulting in defective antigen presentation [34]. In melanocytes, AP-3 functions to deliver the melanin synthesis-related enzyme tyrosinase to melanosomes from endosomes [35] (Figure 1). Thus, HPS2 patients are characterized by partial albinism and signs of immunodeficiency because the AP-3 functions in both the lysosomal compartment in APCs and melanosome in melanocytes.

HPS7 is caused by an alteration in *DTNBP1*, a gene for dysbindin protein [36]. Sandy (sdy) is a mouse variant, which has a deletion of the *Dtnbp1* gene and lack the dysbindin protein expression. Sdy mutant mice are used as an animal model for HSP because sdy mice show hypopigmentation due to melanosomes dysfunction as well as lysosome disfunction and platelet defects [37]. Dtnbp1 is a component of the biogenesis of lysosome-related organelles complex 1 (BLOC-1) and regulates lysosomal organelle transport and biosynthesis. BLOC-1 is known to interact with AP-3 to promote the transport of tyrosinase-related protein 1 (TYRP1) in melanocytes (Figure 1). In addition, BLOC-1 also interacts with the biogenesis of lysosome-related organelles complex 2 (BLOC-2) and promotes melanin synthesis by promoting TYRP1 trafficking by a mechanism different from that of AP-3 [38] (Figure 1). Although there is insufficient evidence that BLOC-1 deficiency is a direct cause of immunodeficiency, AP-3 and BLOC-1 are required for cytokine signaling from plasma membranes and endosomes [39]. In endosomal TLR signaling in plasmacytoid dendritic cells (pDCs), AP-3, BLOC-1, and BLOC-2 are essential for signaling transduction via TLR7 and TLR9, which sense viral nucleic acids and induce type I interferon production [40]. Thus, a dysfunction of lysosome and melanosome-related molecules is responsible for hypopigmentation and immune cell dysfunctions observed in HPS.

### 3.3. Melanin and Inflammatory Responses

It has been reported that the melanin pigment itself may play a role in controlling the immune response. Compared to melanocytes that contain a large amount of melanin, melanocytes containing little melanin produce a higher number of cytokines such as IL-6 and IL-10 [41,42]. l-DOPA, the intermediate product of melanin synthesis, and its oxidation products function as potent immunosuppressive agents that inhibit lymphocyte proliferation and abrogate inflammatory cytokine production by activated lymphocytes [41,42]. Mohagheghpour et al. have shown that non-toxic concentrations of synthetic melanin suppress the production of cytokines such as TNF, IL-1β, IL-6, and IL-10 by reducing the efficiency of mRNA translation [43]. They suggested the possibility of treatment with melanin for pathological symptoms such as rheumatoid arthritis and sepsis syndrome caused by the overproduction of inflammatory cytokines [43]. Individuals with human immunodeficiency virus (HIV) infection often develop oral pigmentation for an unknown reason [44]. In vitro, non-toxic concentrations of melanin inhibit HIV virus-induced syncytia formation and cytopathic effects, with potential therapeutic utility in the treatment of acquired immunodeficiency syndrome (AIDS) [45]. These reports suggest that melanocytes have a role in controlling the immune response and that the presence of melanin pigments may cause fluctuation of the immune response levels.

## 4. Melanogenesis and Toll-Like Receptors

Melanocytes express the innate immune receptors TLRs and directly sense environmental stimuli through TLRs. Human melanocytes express TLR1-4, 6, 7, 9 mRNA, and the expression of TLR2, 3, 4, 7, 9 protein was confirmed in vitro [46]. With stimulation by TLR2, 3, 4, 7, 9 agonists, melanocytes induce the nuclear translocation of NFκB p65 and IκBα phosphorylation, and produce inflammatory cytokines and chemokines including IL-6, IL-8, CCL2, 3, and 5 [46,47]. Gene expressions of TLR1-10 and MyD88 were also observed in melanocytes in the ocular choroid coat [48]. High TLR1-6 protein expression and their functions in melanocytes of the choroid are thought to be a trigger for eye inflammation [48]. Such reports suggest that melanocytes, like other epidermal constitutive cells, have roles in augmenting innate immune inflammatory reactions. Not only are they involved in inflammatory reactions, innate immunity affects melanocytes’ unique role in pigment-producing in the human epidermis. Studies in recent years have suggested that certain innate immune stimuli affect pigment production and the transport of melanosomes (Table 1 and Table 2). In this section, we discuss TLRs 2, 3, 4, 7, and 9, the functional expression of which has been reported in melanocytes.

### 4.1. TLR2 and Melanocyte Functions

**Figure 2:**
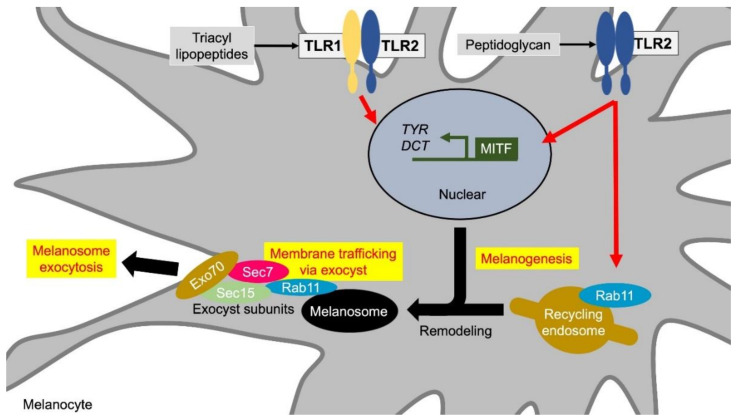
TLR1/2 and TLR2 induce melanin formation and melanosome exocytosis. Stimuli for the TLR1/2 heterodimer and the TLR2 homodimer promote melanin synthesis by inducing the transcription of melanin synthesis-related genes such as MITF, TYR, and DCT. In addition, TLR2 induces extracellular release of melanosomes by increasing the expression of Rab11. Rab11 initiates exocytosis through the remodeling of mature melanosome membranes. Rab11 also constitutes a subunit with tethering factors such as Sec8, Sec15, and Exo70, and anchors melanosomes to the cell membrane. Abbreviations: TLR: toll-like receptor; MITF: microphthalmia-associated transcription factor; TYR: tyrosinase; DCT: dopachrome tautomerase.

TLR2 recognizes a wide range of lipoproteins and lipopeptides of various bacteria including Gram-positive bacteria. TLR2, one of the functional TLRs expressed in melanocytes, induces the genes related to melanin synthesis, and increase the melanin content in cultured human melanocytes [49,50]. TLR2 forms a homodimer receptor complex and heterodimers with TLR1 and TLR6, which expand the range of PAMPs to recognize and diversify intracellular signaling cascades. The TLR2/2 homodimer recognizes bacterial cell components such as Heat-killed *Listeria monocytogenes* (HKLM). The TLR1/2 complex recognizes the lipid composition of native mycobacterial lipoproteins and some triacylated lipopeptides. Pam3CSK4, an agonist of TLR1/2, has been reported to increase melanin synthesis in melanocytes [50,57] (Figure 2, Table 1). Hence, TLR2/6 agonists FSL-1 and Pam2CSK4 did not alter the amount of extracellular melanin [49].

TLR2/2 agonist HKLM increases melanin synthesis, extracellular melanin release, and melanosome transport to adjacent keratinocytes [49]. The formation of skin color requires the transfer of melanin, which is transported out of melanocytes after synthesis, to keratinocytes. Although there are several theories about the melanin transport model, the detailed molecular mechanism of the exo-endocytosis model of melanin, melanocore, and melanosome had long remained obscure [58]. Recent studies revealed that small GTPase Rab11 is involved in the release of melanocytes from melanocytes by exocytosis. Rab11B induces exocytosis through remodeling of the mature melanosomal membrane [59,60]. Exocytosis is initiated by the interaction between Rab11 and tethering factors, which are composed of eight subunits including Sec8, Sec15, and Exo70. Rab11 and the tethering factors anchor melanosome vesicles to the plasma membrane [60]. Rab11A is structurally very similar to Rab11B and interacts with Sec15 in mammals [54,61]. TLR2 agonist HKLM increases melanosome transport to keratinocytes through Rab11A induction [54,61] (Figure 2, Table 2).

The experimental evidence of TLR2-mediated melanin synthesis and transport would explain the possible mechanism of pigmentation after bacterial infection and post-inflammatory hyperpigmentation (PIH). PIH occurs on inflamed skin and is observed more frequently in people of color than in whites [62]. PIH on the face is common in acne, in which *Cutibacterium acnes* exacerbates inflammation. Direct TLR2 stimuli by bacterial components may induce PIH.

### 4.2. TLR3, Viral Infection, and Melanocyte Functions

TLR3 recognizes the double-stranded RNA (dsRNA) of the virus. Human melanocytes express a functional TLR3 receptor and are thought to mount an immediate innate immune response to viruses infecting the skin and epidermis [46]. Recent studies suggest that TLR3-mediated intracellular mechanisms have multiple functions in the skin in addition to the immune response. Studies with TLR3-deficient mice have shown the importance of damage detection by TLR3 in wound healing [63]. Furthermore, TLR3 agonist Poly(I:C) treatment in mice increases the mobilization of neutrophils and macrophages via up-regulation of chemokines and macrophage inflammatory protein-2 (MIP-2/CXCL2), and thus wound healing is promoted [64]. It was also demonstrated that, in human keratinocytes, the activation of TLR3 did not affect the differentiation or growth, but induced the expression of genes involved in the formation of the physical barrier of the skin [65].

**Figure 3:**
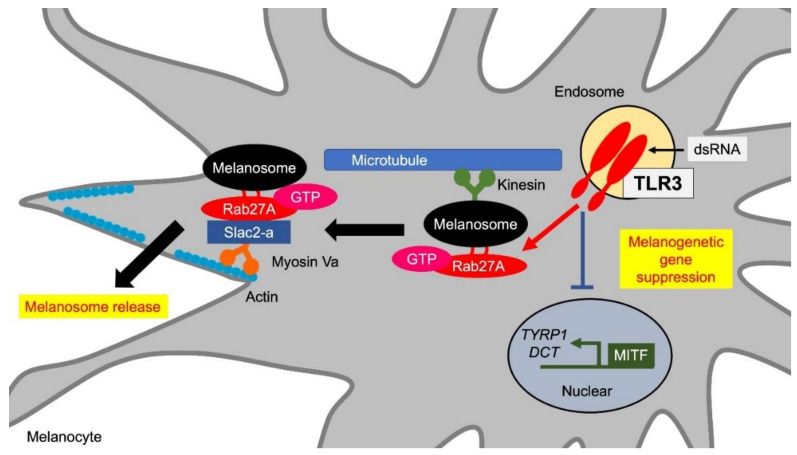
TLR3 promotes melanosome transfer. TLR3 stimulation reduces de novo melanin synthesis by suppressing the expression of MITF, TYRP1, and DCT. On the other hand, TLR3 stimulation promotes extracellular release of melanosomes through Rab27A induction. Rab27A on melanosomes binds to the actin-dependent motor protein myosin Va via Slac2-a and promotes actin transport toward the cell periphery near the cell membrane by forming a tripartite complex. Abbreviations: TLR: toll-like receptor; MITF: microphthalmia-associated transcription factor; TYRP1: tyrosinase-related protein 1; DCT: dopachrome tautomerase.

In the relationship between TLR3 and skin color, multiple reports demonstrated the involvement of viral infections and vitiligo development. Vitiligo is a complex disease caused by a combination of multiple genetic and environmental factors. A large genome-wide association study conducted by Spritz et al. found about 50 distinct loci that contribute to the risk of vitiligo and are implicated in cellular apoptosis and melanocyte function regulation in addition to immunoregulation [66]. From the point of innate immune responses, TLR3-mediated viral stimuli might be involved in the development of vitiligo. In 1996, cytomegalovirus, a type of herpesvirus with double-stranded DNA as its genome, was identified in a skin biopsy specimen of vitiligo patients [67]. Anti-CMV levels in sera of patients with advanced vitiligo are significantly higher than in healthy individuals [68]. Furthermore, a strong association between turkey herpesvirus and vitiligo was suggested in the Smyth line chicken vitiligo model [69]. In addition to herpesviruses, human immunodeficiency virus (HIV), hepatitis B virus, and chronic hepatitis C virus have been suggested to be involved in vitiligo [70,71,72]. A high concentration of double-stranded RNA, which is generated as an intermediate metabolite during viral replication, has been shown to cause apoptosis in melanocytes due to TLR3-mediated IFNγ autocrine signaling [73]. We have reported that TLR3 stimulation by non-toxic doses of viral double-stranded synthetic RNA Poly(I:C) suppressed de novo melanin synthesis in melanocytes [49] (Figure 3, Table 1). These observations indicate that viral infection and its resulting TLR3 stimuli would contribute to the vitiligo pathogenesis not only by melanocyte apoptosis but also by melanocyte dysfunction, namely the suppression of melanin synthesis.

The question arising here is why viral infections induce the inhibition of melanogenesis in the skin. A report from Harson et al. provides one important hint. They showed that melanocytes could be a primary site of viral replication in the skin in the early stages of viral infection [74]. Varicella-zoster virus (VZV) is a member of the herpesviridae family. Congenital varicella syndrome, which rarely occurs in the fetus due to VZV, is known to cause depigmentation around scarred skin [75]. In the process of elucidating the pathways of VZV envelopment and egress, they showed that melanocytes may have a favorable cytosol for VZV replication. They pointed out some commonalities between VZV virion formation and melanosome formation in melanocytes: premelanosome formation, melanosome maturation process, and the extracellular release mechanism of melanosome. Harson et al. proposed the hypothesis that VZV deprives a melanocyte of its cellular mechanism [75]. Given that the intracellular mechanism of melanocyte pigment formation is utilized and hijacked for virus replication, pigment formation in melanocytes would then be inhibited. TLR3 signaling takes place intracellularly and requires endosome maturation [76]. In monocyte-derived immature dendritic cells, TLR3 is mainly localized intracellularly on the endosome, and viral molecules are transported to TLR3 through endocytosis. Studies with anti-TLR3 mAbs have shown that fibroblast TLR3 acts on the cell surface to detect viral infections, and endosome maturation is required to evoke TLR3 signaling intracellularly [76]. Because endosomes are transporting cargo to lysosomal organelles such as melanosomes and polyendoplasmic reticulum [77], utilization of endosomes for viral TLR3 signaling may suppress melanosome maturation and melanin synthesis. Alternatively, the viral RNA carried into the cytosol may be transported into the pre-melanosomes, resulting in melanosome dysfunction.

During the suppression of de-novo melanin synthesis by TLR3 stimuli, we also observed that TLR3 agonist Poly(I:C) increased Rab27A expression in melanocytes and induced melanosome translocation from the perinuclear to the cell periphery close to membrane [49] (Figure 3, Table 2). Rab27A is a low molecular weight G protein localized on melanosomes in melanocytes, and secretory granules in cytotoxic T cells and endocrine cells [78]. In melanocytes, two effectors, Slp2-a/melanophilin (synaptotagmin-like protein 2-a) and Slac2-a (Slp homologue lacking C2 domains), specifically bind to active GTP-Rab27A [79]. Actin transport proceeds by binding Rab27A on melanosomes and myosin Va, which is an actin-dependent motor protein, via Slac2-a to form a tripartite complex. After actin transport, melanosomes anchor to the plasma membrane by the binding of Rab27A on the melanosomes to phosphatidylcholine in the cell membrane via Slp2-a [79]. Poly(I:C) is thought to regulate melanosome transport by anchoring melanosomes to the cell membrane and promoting extracellular release through an increase in Rab27A [49].

A novel identification of the close relationship between UV irradiation and TLR3 may provide new insight into UV-dependent skin pigmentation. Self-noncoding RNA produced by UVB exposure activates TLR3 and induces inflammatory cytokine release from keratinocytes and peripheral blood mononuclear cells [80]. The promotion of TLR3-mediated skin barrier repair gene expression is also essential for restoration of the skin permeability barrier function after UVB injury [81]. Following a report that TLR3 is essential for UVB exposure-induced immune reactions and skin barrier recovery, we revealed that UVB irradiation enhances melanosome transport by Rab27A via TLR3 similarly to the stimulation by TLR3 agonist Poly(I:C) [49]. Therefore, melanocyte TLR3 functions as a modulator of skin pigmentation not only by viral molecule stimuli but also by UV irradiation to promote melanosome transfer to keratinocytes.

Intracellular melanosome transport occurs not only by Rab27A, TLR3 stimulation also increases the extracellular release of melanosomes via Rab11, which induces melanocyte exocytosis as does TLR2 stimulation [54] (Table 2). Since keratinocytes also express both TLR2 and TLR3 as melanocytes do, it is a very interesting question how the innate immune stimuli modulate melanosomes and melanin in keratinocytes after being transferred from melanocytes. We observed that TLR3 stimulation induces melanin uptake by activating keratinocyte phagocytosis [55]. We also showed that stimulation of TLR3 agonist Poly(I:C) upregulates keratinocyte melanosome uptake by controlling the expression and activity of factors related to phagocytosis, including protease-activated receptor 2 (PAR2) and RHO family members RHOA and CDC42. It is known that PAR2 is induced by UV irradiation in keratinocytes and promotes the phagocytosis of keratinocytes [82,83] (Table 2). The RHO family GTP-binding proteins are involved in cytoskeletal remodeling during phagocytosis and are major factors controlling endocytosis and phagocytosis [84]. Thus, innate immune stimulation affects keratinocyte melanin uptake and controls pigmentation by activating the phagocytosis of keratinocytes.

Melanin and melanosome in keratinocytes undergo degradation as keratinocyte differentiation progresses. Although the mechanism behind this is not fully understood, TLR3 innate immunity is likely to be involved in the melanin degradation. The TLR3 agonist Poly(I:C) activates autophagy in cultured human keratinocytes [56]. In turn, activated autophagy suppressed the Poly(I:C)-stimulated inflammatory response in keratinocytes [56]. Not only controlling inflammatory responses, of note, autophagy is responsible for melanin degradation in keratinocytes during keratinocyte differentiation [85,86]. In keratinocytes, suppression of the function of Heat shock protein 70 (Hsp70) induced the degradation of incorporated melanosomes. Studies with Hsp70 inhibitors showed increased levels of LC3-II that correlated with autophagy activation within keratinocytes [87]. Since the heat shock response and autophagy have opposite homeostatic systems of intracellular protein synthesis and degradation, Hsp70 is thought to be involved in a negative regulatory feedback loop that reduces cellular autophagy [88]. Autophagy is an action that maintains intracellular homeostasis by eliminating aged and damaged organelles. Autophagy maintains cell survival under conditions with high external stress such as hypoxia, oxidative stress, bacterial and microbial infections [89]. Autophagy is also involved in immunity such as the elimination of microorganisms and participation in antigen presentation [90]. In human epidermis, autophagy affects keratinocyte differentiation and melanocyte survival in epidermal cells, and is associated with the pathogenesis of various skin diseases including psoriasis, systemic lupus erythematosus, and vitiligo [91]. Therefore, TLR3 would be involved in melanin degradation in keratinocytes through an autophagy mechanism.

### 4.3. TLR4 and Melanocyte Functions

**Figure 4:**
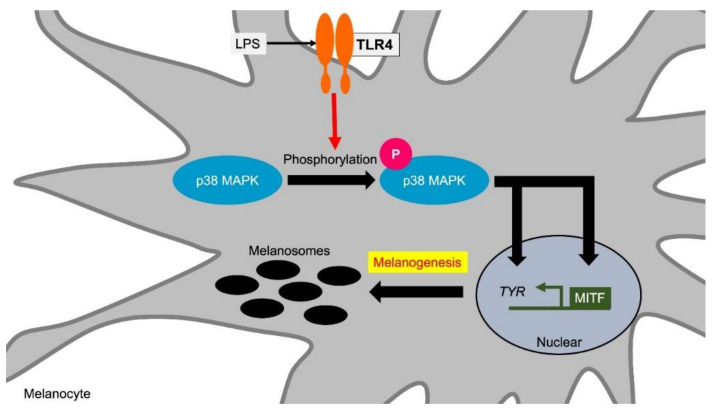
TLR4 induces melanogenesis via p38 MAPK. TLR4 stimulation increases melanin synthesis by inducing the expression of TYR and its transcription factor MITF. TYR and MITF induction are mediated via p38 MAPK phosphorylation by TLR4 stimuli. Abbreviations: TLR: toll-like receptor; MITF: microphthalmia-associated transcription factor; TYR: tyrosinase; MAPK: mitogen-activated protein kinase; LPS: lipopolysaccharide.

The relationship between the TLR-mediated immune response and melanogenesis is the most studied feature of TLR4. TLR4 recognizes lipopolysaccharide (LPS) derived from Gram-negative bacteria. TLR4 is also suggested to be involved in the pathogenesis of autoimmune diseases such as rheumatoid arthritis, systemic lupus erythematosus and skin sclerosis [92]. In melanocytes, the expression of TLR4 and its adapter molecules CD14 and MyD88 has been confirmed in cultured human melanocytes. The TLR4 agonist lipopolysaccharide (LPS) increases the expression of TLR4, CD14 and MyD88, and induces pigmentation accompanied by inflammatory cytokine induction [47]. TLR4 activation by LPS induces the expression of tyrosinase and its transcription factor MITF in melanocytes via the activation of p38 MAPK (mitogen-activated protein kinase) [51] (Figure 4, Table 1).

In human neonatal melanocytes, repeated UV irradiation increases TLR4 expression and pigmentation [93]. LPS stimulation with repeated UV irradiation induces IL-6 secretion from human neonatal melanocytes, suggesting that melanocytes participate in UV-induced immune modulation [93]. Indeed, TLR4 causes a reaction similar to UV irradiation in Langerhans cells, suggesting that TLR4 is involved not only in bacteria recognition but also in an immune regulation similarly to UV-induced cutaneous immune regulation [94]. Candidiasis is an infectious disease that accounts for more than half of all systemic mycoses. TLR4 can recognize *Candida albicans* infection and shows protective effects against candida infection. In human melanocytes, *Candida albicans* induces pigment production via TLR4, by which melanocytes reduce the infectivity of *Candida albicans* [95]. Dark-colored melanocytes have higher levels of TLR4 expression and different levels of inflammatory response compared to light-colored melanocytes [41]. This suggests that the melanocyte pigment levels influence the magnitude of the inflammatory response in human skin. These examples indicate that pigmentation and its process by TLR4 stimulation play roles in immune defenses and inflammatory responses.

### 4.4. TLR7 and Melanocyte Functions

**Figure 5:**
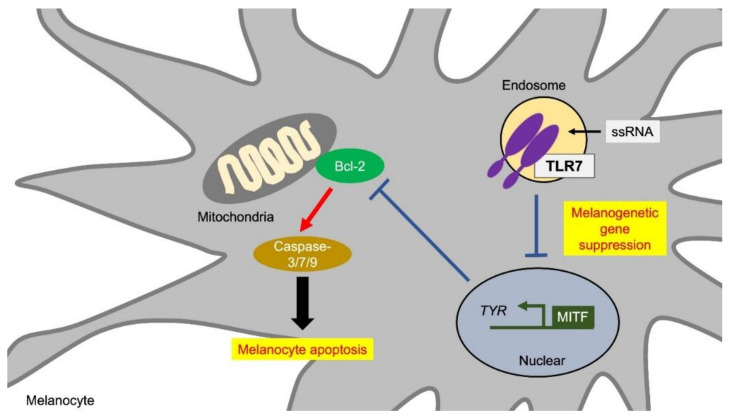
TLR7 inhibits melanogenesis and induces melanocyte apoptosis. TLR7 stimulation induces pigment loss due to two factors: decrease in the expression of melanin synthesis-related genes and the induction of apoptosis. TLR7 stimulation suppresses the expression of MITF and TYR. The decrease in MITF suppresses Bcl-2 expression in melanocytes. Bcl-2 is localized in mitochondria and has an anti-apoptotic function. Therefore, the decrease in MITF leads to apoptosis through caspase activation by Bcl-2 suppression. Abbreviations: TLR: toll-like receptor; MITF: microphthalmia-associated transcription factor; TYR: tyrosinase.

TLR7 is localized in intracellular organelles, recognizes single-stranded ssRNA derived from a virus, and induces an antiviral response such as interferon production via MyD88 in dendritic cells. In human melanocytes, the signals mediated by TLR7 are suggested to suppress melanin production. Imiquimod, a synthetic low-molecular-weight agonist for TLR7, is used as a therapeutic drug for condyloma acuminata and carcinoma in situ. Imiquimod induces apoptosis of melanocytes as well as keratinocytes and fibroblasts [96,97]. Topical administration of imiquimod occasionally induces vitiligo-like hypopigmentation [50,98]. A decrease in the pigment level on the body surface was also confirmed in a zebrafish model system [50,98]. A genetic association analysis for vitiligo revealed susceptibility loci including TLR7 single nucleotide polymorphisms (SNPs) [99]. Melanocytes collected from vitiligo lesions show an increase of TLR7 and TLR9 expression along with an increase of the apoptosis level and a decrease of the melanin synthetic ability [100]. In vitro studies have shown that imiquimod suppresses the expression of MITF and tyrosinase in human melanocytes [52] (Figure 5, Table 1). Since MITF induces the expression of Bcl-2, which is localized in mitochondria and has an anti-apoptotic function, the suppression of MITF by imiquimod would reduce Bcl-2 and cause apoptosis through caspase activation [101] (Figure 5). Thus, TLR7 stimuli induce depigmentation by the increase of melanocyte apoptosis and by the suppression of the melanogenic gene expression.

### 4.5. TLR9 and Melanocyte Functions

TLR9 recognizes unmethylated CpG DNA from bacteria and viruses. TLR9 also recognizes nucleic acids from host cells and tissues to induce interferons through the NFκB pathway [102,103].

**Figure 6:**
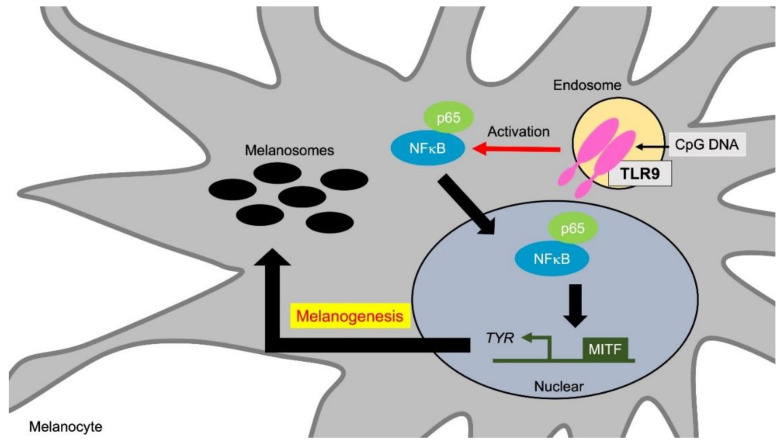
TLR9 promotes melanogenesis via NFκB activation. TLR9 stimulation increases melanin synthesis via TYR and PMEL induction through NFκB activation. TYR and PMEL induction by TLR9 stimulation is further enhanced by the synergistic effect with UVB irradiation. Abbreviations: TLR: toll-like receptor; TYR: tyrosinase; MITF: microphthalmia-associated transcription factor; PMEL: premelanosome protein.

Human melanocytes express functional TLR9, and TLR9 ligands activate the NFκB signaling and induce cytokines and chemokines expression from melanocytes [46]. Cytosine guanine ODN2006, an agonist of TLR9, increases melanin synthesis via NFκB activation [53] (Figure 6, Table 1). The combination of ODN2006 and UVB irradiation significantly increased the expression of tyrosinase and PMEL in melanocytes [53]. Therefore, TLR9 stimulation tends to enhance melanogenesis along with inflammatory cytokine induction in human melanocytes.

As described in the section on TLR7, increased expression of TLR7 and TLR9 are observed in melanocytes obtained from vitiligo lesions [100]. Although TLR7 and TLR9 are similar in structure and involved in autoimmunity by recognizing self-RNA and self-DNA, respectively, contradictory actions for autoimmune disease models are observed. For example, TLR9-deficient mice showed exacerbated symptoms in a mouse model of systemic lupus erythematosus, while attenuated symptoms were observed in TLR7-deficient mice [104]. The TLR trafficking chaperone UNC93B1, which prevents autoimmunity through the recruitment of syntenin-1, suppresses TLR7 specifically without affecting TLR9 [105]. Since the vitiligo pathology has an aspect of an autoimmunity against melanocytes, it is possible that the increased expression of these two TLRs may be due to competition for maintaining homeostasis in the vitiligo lesions.

## 5. Future Prospect for Studies of Melanogenesis in Immune Systems

Studies on innate immune systems have revealed that the TLR-mediated intracellular mechanism has multiple functions in melanogenesis. Since the melanin pigment itself has a role in the immune response, it is reasonable to consider that innate immune stimulation controls pigment formation. Exploring the role of melanin pigments in the immune response may lead to understanding how melanocytes and melanogenesis evolved to acquire their regulatory mechanisms and functions. Studies of the TLRs involvement in melanogenesis have only just been launched.

At present, little is known about how the TLRs affects the dynamics of melanosome and melanin in keratinocytes. Keratinocytes express functional TLRs. The link between TLRs and lysosome are extensively studied in the field of acquired and innate immunity. Therefore, TLRs might affect the melanosome degradation through lysosome and autophagy activation in keratinocytes. In the future, the involvement of immune signals in pigment formation should be explored to understand the cycle of melanin synthesis, transport, uptake, and degradation in keratinocytes, which are essential molecular actions controlling the skin color. The development of such studies will promote the understanding of the biological defense involving pigmentation control in the living body and elucidate the mechanisms of immune dysfunction in skin diseases with pigment abnormality.

## Figures and Tables

**Table 1:** Toll-like receptors (TLR) expression in melanocytes and the effect of each ligand on melanogenesis.

TLR	TLR ligands	TLR Expressions in Melanocytes		Effect of TLR Ligands on Melanogenesis		
		Protein	mRNA	Melanogenesis	Targeted Genes	References
TLR1	Triacyl lipopeptides	N.D.	✓	N.E.		[46]
TLR2	Peptidoglycan	✓	✓	Promote	TYR, DCT, MITF	[46,49]
TLR3	dsRNA	✓	✓	Inhibit	TYRP1, DCT	[46,49]
TLR4	Lipopoly-saccharide	✓	✓	Promote	TYR, MITF, DCT	[46,47,49,50,51]
TLR5	Flagellin	N.D.	N.D.	Inhibit	Unknown	[46,50]
TLR6	Diacyl lipopeptides	N.D.	✓	N.E.		[46]
TLR7	ssRNA	✓	✓	Inhibit	TYR, MITF	[46,50,52]
TLR8	ssRNA	N.D.	N.D.	N.E.		[46]
TLR9	CpG-DNA	✓	✓	Promote	TYR, PMEL	[46,53]

TLR; toll-like receptor, TYR; tyrosinase, DCT; dopachrome tautomerase, MITF; microphthalmia-associated transcription factor, TYRP1; tyrosinase related protein 1, PMEL; premelanosome protein, ✓; detected in melanocytes, N.D.; not detected, N.E.; not examined.

**Table 2:** Effect of innate immune stimulation on melanin transfer from melanocyte to keratinocytes.

Action			TLR2	TLR3		
**Melanin Transfer**	in MC	Effect	N.E.	Promote		
		Targeted molecule		Rab27A		
		Ref.		[49]		
**Melanin Release**	from MC	Effect	Promote	Promote		
		Targeted molecule	Rab11A	Rab11A		
		Ref.	[54]	[54]		
**Melanin Uptake**	by KC	Effect	N.E.	Promote		
		Targeted genes		PAR-2	RHOA	CDC42
		Ref.		[55]		
**Melanin Degradation**	in KC	Effect	N.E.	Promote		
		Targeted molecule		Autophagy activation		
		Ref.		[56]		

MC; melanocytes, KC; keratinocytes, TLR; toll-like receptor, PAR-2; protease- activated receptor-2, RHOA; Ras homolog family member A, CDC42; cell division control protein 42 homolog, N.E.; not examined, Ref.; References.
